# Ultrasound-assisted lumbar puncture with a horizontal and perpendicular paramedian approach based on positioning in patients with abnormal spinal anatomy: a case report and technical description

**DOI:** 10.1186/s12871-023-02368-7

**Published:** 2023-12-12

**Authors:** Chanyan Huang, Jiawen Li, Yuting Guo, Yuanjia Zhang, Wei Luo, Daniel A. Diedrich, Tao Zhang, Wenqi Huang, Ying Xiao

**Affiliations:** 1 Department of Anesthesiology, The First Affiliated Hospital, Sun Yat-sen University, No. 58, Zhongshan 2nd Road, 510080 Guangzhou, China; 2 Department of Neurology, Peking Union Medical College Hospital, Beijing, China; 3 Department of Anesthesiology and Perioperative Medicine, Mayo Clinic, Rochester, MN USA

**Keywords:** Lumbar puncture, Spinal anesthesia, Scoliosis, Ultrasonography, Case report

## Abstract

**Background:**

The use of ultrasound has been reported to be beneficial in challenging neuraxial procedures. The angled probe is responsible for the main limitations of previous ultrasound-assisted techniques. We developed a novel technique for challenging lumbar puncture, aiming to locate the needle entry point which allowed for a horizontal and perpendicular needle trajectory and thereby addressed the drawbacks of earlier ultrasound-assisted techniques.

**Case presentation:**

Patient 1 was an adult patient with severe scoliosis who underwent a series of intrathecal injections of nusinersen. The preprocedural ultrasound scan revealed a cephalad probe’s angulation (relative to the edge of the bed) in the paramedian sagittal oblique view, and then the probe was rotated 90° into a transverse plane and we noted that a rocking maneuver was required to obtain normalized views. Then the shoulders were moved forward to eliminate the need for cephalad angulation of the probe. The degree of rocking was translated to a lateral offset from the midline of the spine through an imaginary lumbar puncture’s triangle model, and a needle entry point was marked. The spinal needle was advanced through this marking-point without craniocaudal and lateromedial angulation, and first-pass success was achieved in all eight lumbar punctures. Patient 2 was an elderly patient with ankylosing spondylitis who underwent spinal anesthesia for transurethral resection of the prostate. The patient was positioned anteriorly obliquely to create a vertebral rotation that eliminated medial angulation in the paramedian approach. The procedure succeeded on the first pass.

**Conclusions:**

This ultrasound-assisted paramedian approach with a horizontal and perpendicular needle trajectory may be a promising technique that can help circumvent challenging anatomy. Larger case series and prospective studies are warranted to define its superiority to alternative approaches of lumbar puncture for patients with difficulties.

## Introduction

Preprocedural ultrasound has been demonstrated to be beneficial for lumbar puncture (LP) in patients with complex spinal anatomy [1, 2]. However, technical limitations such as the inaccuracy of skin markings and dependence on the memory to replicate the estimated angle of the probe during needle insertion may significantly affect its applications [3, 4]. Therefore, we designed a novel ultrasound-assisted approach for LP in challenging patients, focusing on eliminating the need for probe angulation in both transverse and sagittal views, to allow for a horizontal and perpendicular needle trajectory. We report a series of successful LPs using this novel technique in a patient with severe scoliosis and another patient with ankylosing spondylitis.

## Case presentation

### Patient 1

**Fig. 1:**
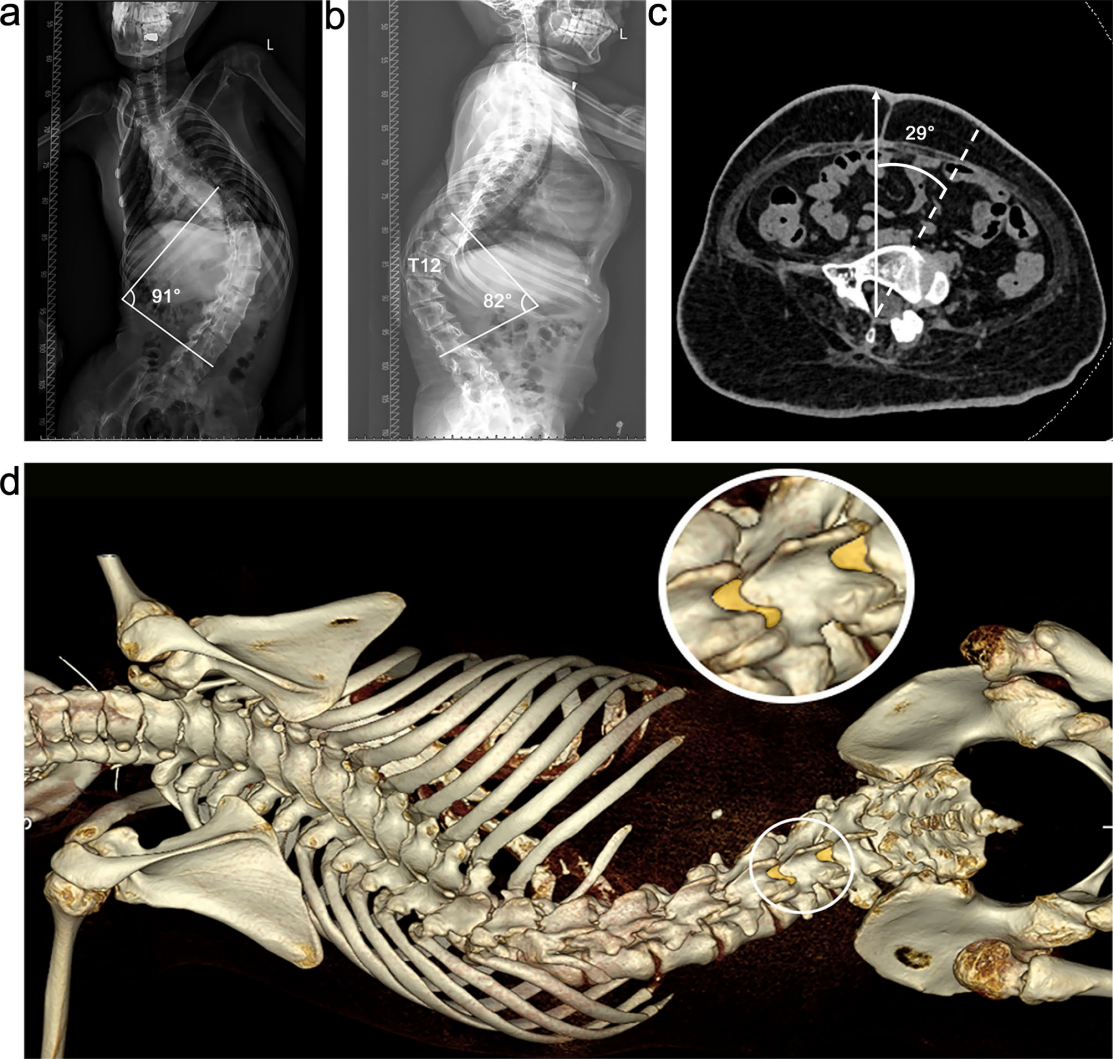
Radiographic evaluation of the spine (Patient 1). (**a**) The anterior-posterior radiographic view demonstrating thoracolumbar scoliosis with a Cobb angle of 91°. (**b**) Lateral radiography demonstrating thoracolumbar kyphosis with a Cobb angle of 82°. (**c**) Axial CT view demonstrating L5 vertebral body rotation calculated by the angle at which the vertebral (white, broken line) was inclined relative to a vertical line (white, unbroken arrow). (**d**) Three-dimensional CT of the spine showing capacious L3/4 and L4/5 interspaces on the left side (white circle)

An adult patient, with a diagnosis of spinal muscular atrophy (SMA) type 2 and severe scoliosis, was referred to the Department of Anesthesiology for intrathecal nusinersen administration because of previously failed attempts to perform LP in the Neurology Department. Radiographs showed a lumbar curve toward the left with a Cobb angle of 91° and a profound thoracic kyphosis with a Cobb angle of 82°. The rotation angle of the L5 vertebral bodies was 29°. The capacious L3/4 and L4/5 interspaces were discovered via three-dimensional (3D) computed tomography (CT) (Fig. 1).

**Fig. 2:**
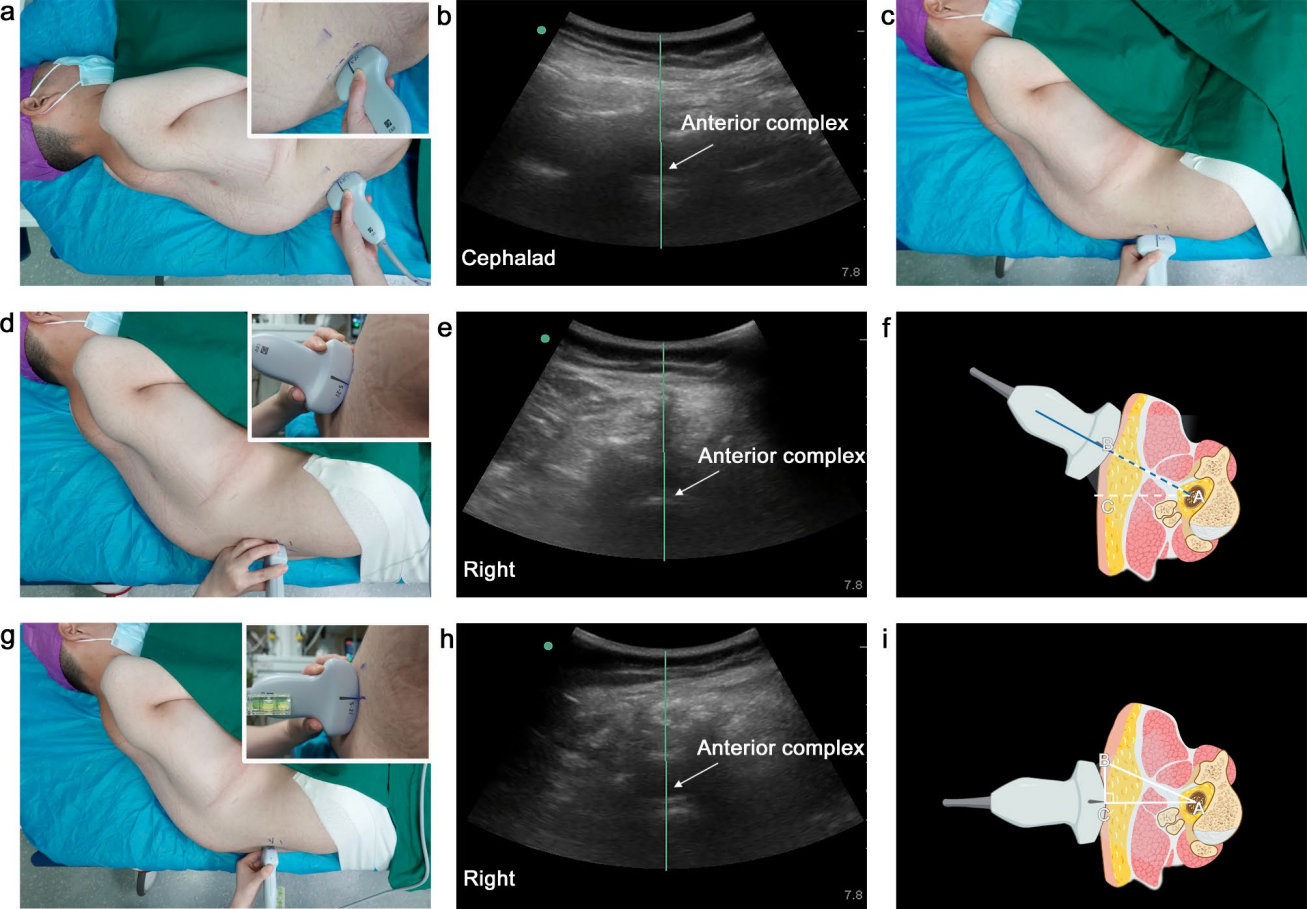
Angle-free paramedian approach for lumbar puncture in a patient with severe scoliosis (Patient 1). (**a-b**) Cephalad angulation of the probe required to obtain a clear anterior complex image in the paramedian sagittal oblique view in the standard lateral decubitus position. (**c**) Modified position to eliminate the cephalad angulation of the probe required for the same image. (**d-e**) The probe was rotated 90 degrees to obtain the transverse median view, and then rocked clockwise (from caudal-to-cephalic view) to produce a normalized transverse interspinous view with the laminae at the same horizontal level. (**f**) Schematic image illustrates the degree of the rocking is the angle by which the trajectory of needle insertion must be modified to successfully direct the needle towards the spinal canal. (**g-h**) The probe was placed parallel to the floor, as verified by attaching a spirit level to obtain the image of the spinal canal. Once the spinal canal is centered in the middle of the screen, skin marks are made at the midpoint of the probe’s long and short edges. The intersection of these two marks is identified as the needle entry point. (**i**) Lumbar puncture’s triangle model. The hypotenuse (AB) is the midline approach; the base (AC) is the paramedian approach, and the height is the lateral offset from the midline approach

Due to neuromuscular disorder, the patient was unable to sit independently and was placed in a standard left lateral decubitus position, with the shoulders and hips vertically aligned with the edge of the bed (Fig. 2a). A preprocedural ultrasound scan was performed using a low-frequency (5 − 2 MHz) curvilinear transducer (Fujifilm Sonosite, Inc., Bothell, WA, USA), according to a scan protocol described previously by Karmakar [5]. Firstly, the probe was placed parallel to the spinous process (SP) line and the interlaminar space was identified by counting upwards from the sacrum in the paramedian sagittal oblique (PSO) view. L4/5 with the clearest and longest anterior complex (AC) was identified as the target interspace. We noticed that the probe has a cephalad orientation, which implies that the posterior aspect of the vertebral body is oriented anteriorly obliquely relative to the edge of the bed (Fig. 2a-b). Consequently, we pushed the shoulders forward and pulled the hips backward to align the posterior aspect of the L5 vertebral body with the edge of the bed as confirmed by the perpendicularly placed probe (Fig. 2c).

Then the transverse scan is performed by positioning the probe perpendicular to the long axis of the spine. To obtain a normalized transverse view, a rocking maneuver was required which indicates an angled median approach (Fig. 2d-f). To make the angle of needle insertion easier to grasp, we introduced a LP’s triangle model to convert this angled approach to a horizontal path right to the center of the thecal sac. The right triangle’s hypotenuse is the angled median approach, while its base is the horizontal paramedian approach and its height is the lateral offset from the midline. To locate this needle entry point, the probe was held perpendicularly to the edge of the bed at the marked interspace, parallel to the floor as verified by a spirit level attached on the probe, with the dural sac centering on the ultrasound screen (Fig. 2g-i). The intersection of the midpoints of the probe’s long and short edges was used as the needle entry point. After local infiltration, a 22-gauge spinal needle was inserted horizontally and perpendicularly to the edge of the bed. There was only one needle pass with a 30-second procedure time (defined as the time from first spinal needle contact to the skin to visualization of the outflow of cerebrospinal fluid) and nusinersen was successfully injected intrathecally. In accordance with the recommended dosing schedule, the nusinersen injection was performed on treatment days 0, 14, 28, and 63 (four loading dosing) followed by maintenance doses every 4 months thereafter for this patient. Therefore, this patient experienced a total of eight lumbar punctures during a period of 18 months. Using this approach of horizontal and perpendicular needle trajectory, first-pass success and first-attempt success was achieved during the following seven nusinersen administrations. For all the eight lumbar punctures, the median locating time (from when the probe was first placed on the skin until the skin marking was complete) was 10.8 min [IQR, 7.8–13.7 min; range, 6 to 17 min], with a procedure time of 36 s [IQR, 30–42 s; range, 28 to 60 s]. The median depth of needle insertion was 5.3 cm [IQR, 5.2–5.5 cm; range, 5.1 to 5.5 cm]. No adverse events were observed during the procedure and within three post-dural puncture days. Both the patient and the proceduralists expressed satisfaction with the ease of injection.

### Patient 2

**Fig. 3:**
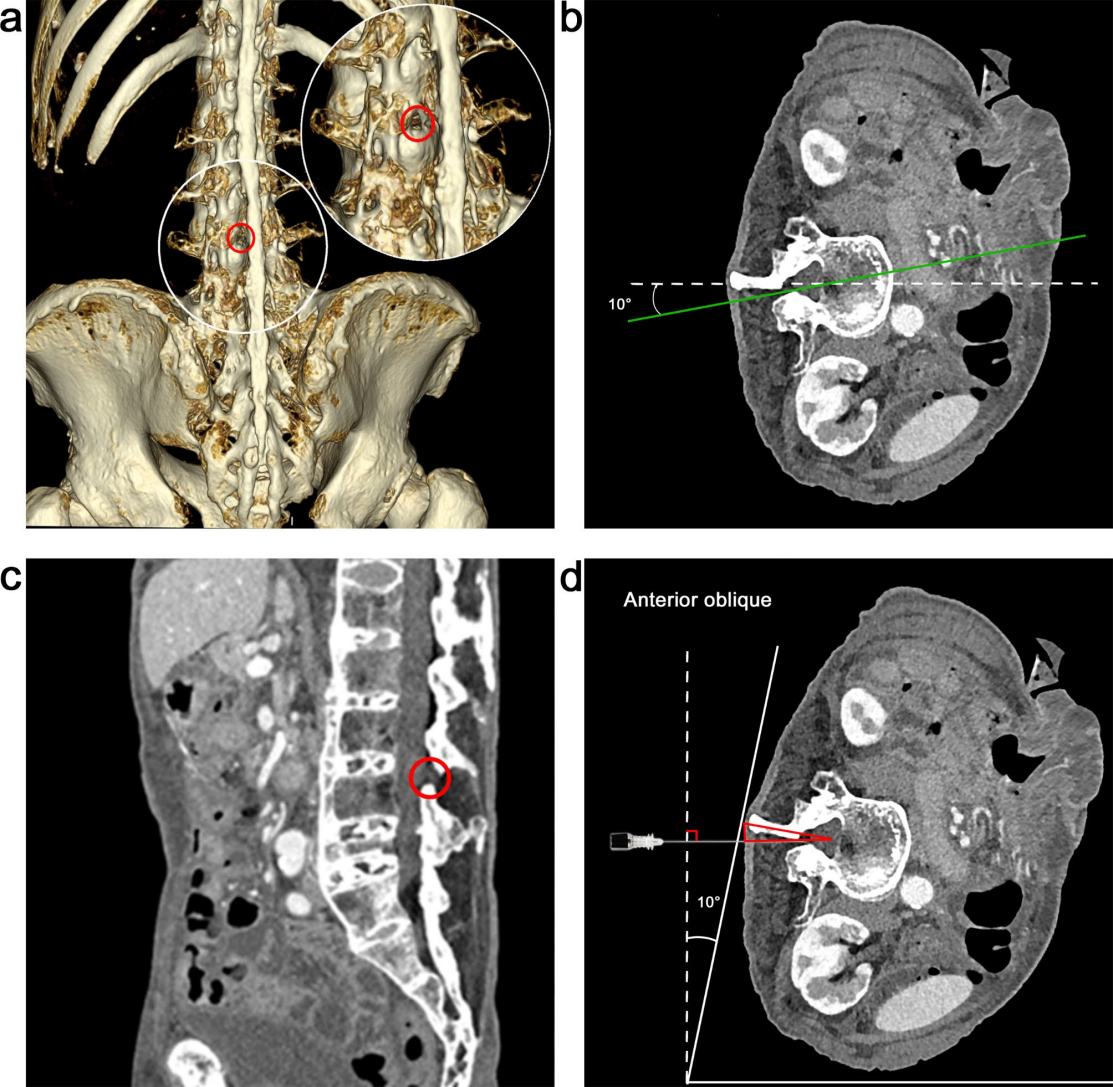
Ultrasound-assisted angle-free paramedian approach in an elderly patient with ankylosing spondylitis (Patient 2). (**a**) Three-dimensional CT image from the posterior view with right rotation, demonstrating narrowing of the interspaces due to extensive ossification and osteoarthritis with patent left L4/5 interlaminar space (red circle). (**b-c**) Axial CT demonstrating the potential needle trajectory of the standard paramedian approach with 10° medial angulation. A sagittal oblique reconstruction along the conventional paramedian needle trajectory reveals a needle trajectory through the interlaminar space without craniocaudal angulation. (**d**) Axial CT demonstrating the design of the horizontal needle trajectory through interlaminar space when the back is lying forward by 10°

Patient 2 was an elderly patient with ankylosing spondylitis who underwent spinal anesthesia for transurethral resection of the prostate to avoid difficult airway management. CT revealed extensive calcification of supraspinous and interspinous ligaments with obliterated interspinous space and most of the interlaminar spaces being obstructed by facet joint osteoarthritis. A patent left L4/5 interlaminar space was found in 3D CT and axial imaging (Fig. 3a-b). Using an oblique sagittal reconstruction via the conventional paramedian route with 10° medial angulation, a needle path through interlaminar space was seen perpendicular to the sagittal plane (Fig. 3b-c). This indicated that if the back was tilted by 10°, the vertebrae were manually rotated and a lumbar puncture’s triangle was created, allowing for a horizontal needle trajectory to direct towards the central part of the sac from the lateral point of entry (Fig. 3d). Therefore, the patient, who was initially placed with standard lateral decubitus position, was then rotated by 10° anterior oblique to induce “vertebral rotation”. After identifying the chosen interspace on the PSO scanning, the probe was held horizontally and perpendicularly to the edge of the bed on the transverse scanning to bring the sac to the center of the screen. The needle entry point was determined at the center of the probe. A total of 5 min was required for the patient positioning and preprocedural ultrasound scanning. At the designated site, a second-year anesthesia resident inserted a 22-gauge (90 mm) spinal needle without craniocaudal and lateromedial angulation and easily penetrated the intrathecal space on the first pass, with a procedure time of 39 s. No procedure-related complications occurred.

## Discussion and conclusions

We describe here a novel ultrasound-assisted paramedian approach to LP that may be applicable to patients with challenging anatomies. In our method, appropriately positioning under ultrasound guidance and utilizing vertebral rotation, either inherent in the scoliotic spine or manually created in the normal spine, are the key modifications to convert the angled conventional approach into an angle-free puncture.

Reducing the technical difficulty of LP is desirable because multiple needle insertion attempts can be distressing for any patient and increase the risk of complications [6]. Evidence supports that ultrasound-assisted technique facilitates LP in the elderly and those with spinal deformities [1, 2, 7, 8]. However, the remained low first-pass success rate even with the aid of preprocedural ultrasound assistance, necessitated the development of a new technique [1–3, 8]. Therefore, we devised this novel technique to address the shortcomings of previous methods.

**Fig. 4:**
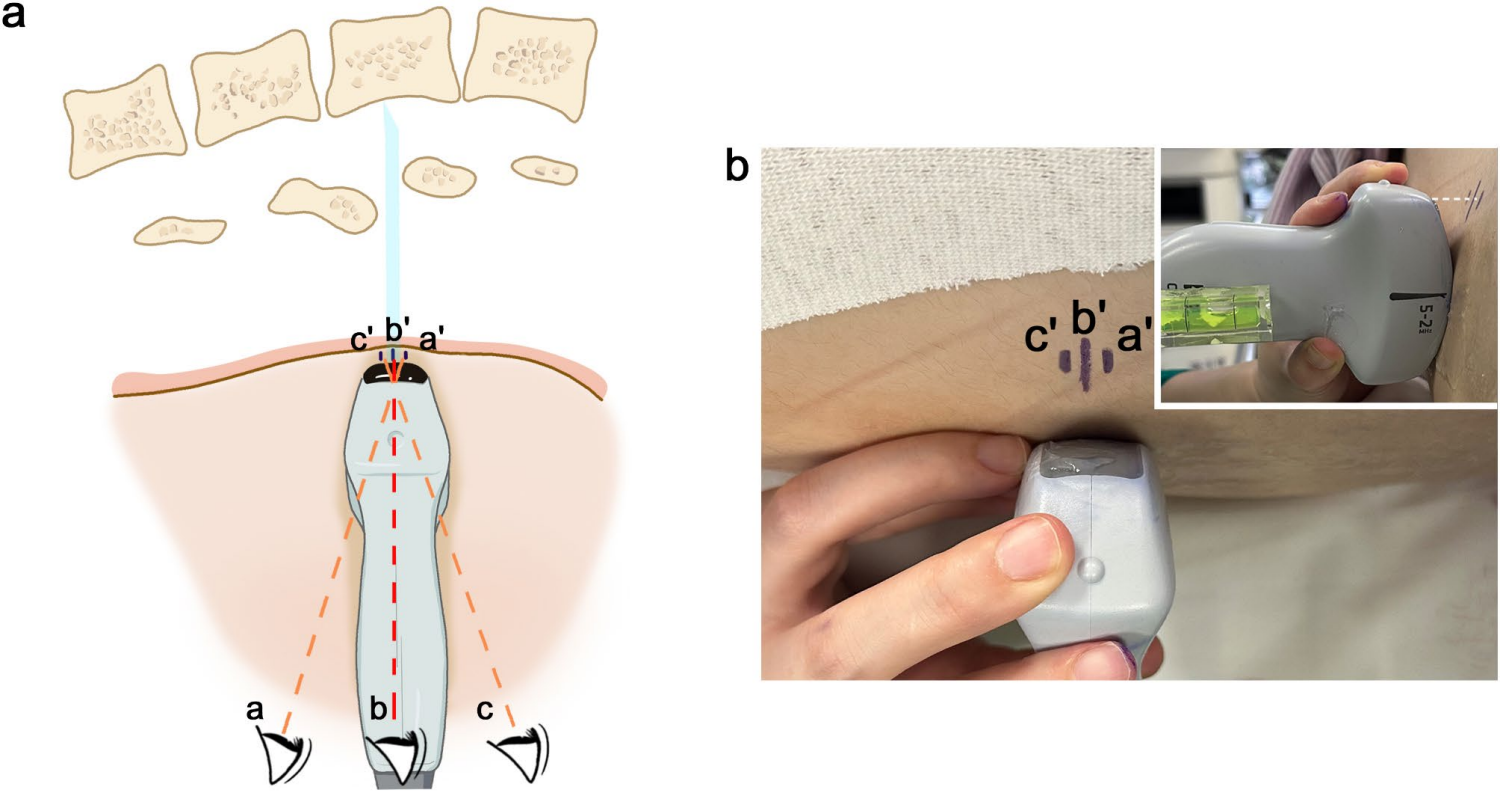
Marking error due to parallax error. Parallax error in marking the midpoint of the short border of the probe (a′ or c′) occurs when the eyes are not in line with the angle of the probe (**a** or **c**), particularly when the probe is not in direct contact with the skin surface (white dotted line). To minimize skin marking inaccuracies (b′), the operator should move the head to keep the line of sight parallel to the ultrasound beam plane (**b**)

First, one of the main limitations of previous ultrasound-assisted techniques was the inaccuracy of skin markings. When a tilt or a rocking maneuver was required to obtain an optimal image, it is difficult to locate the center of the probe [9]. If the probe is tilted and not in direct contact with the skin surface of the lean patients, a marking error on locating the midpoint of the short sides of the curvilinear probe will occur due to parallax when the markings are made from different angles instead of along the incident beam (Fig. 4). Previous studies stated that experience with the ultrasound-assisted technique could compensate for this limitation [4]. However, this experience can only be accumulated through proper basic training. It is easy to inadvertently tilt or rock the probe even when we pay attention to it. Therefore, we attached a spirit level to the probe to visually confirm that it was horizontally placed, so as to improve the precision of skin markings.

Second, accurate measurement and duplication of the angle of the probe during needle insertion are challenging. If the angle at which the spinal needle is inserted differs from the angle used at scanning, though small in magnitude, redirections and attempts may be required in patients with narrowed interlaminar spaces. Notably, the needle insertion angles that anesthesiologists are most familiar with are horizontal and perpendicular. Therefore, we introduced this standardizing needle trajectory to decrease the number of redirections through positioning and triangle model.

We could classify patients with difficult lumbar punctures into two categories based on the presence or absence of vertebral rotation. For patients with vertebral rotation which is most observed in scoliotic spines, we can make use of the vertebral rotation to allow for a horizontal needle trajectory to enter the center of the sac with no medial angulation via the paramedian approach as previously reported [10]. For patients with no vertebral rotation but with an obstructed interspinous route due to degenerative pathology, we can create vertebral rotation through positioning to achieve the paramedian horizontal approach. Tilting the back forward artificially rotates the vertebrae, which allows the needle to be directed towards the central part of the sac from a lateral point of entry horizontally. Using this strategy, we can avoid directing the needle tip to the lateral part of the thecal sac, or even the space between the sac and the nerve root exit, with an increased risk of a “dry tap”.

**Fig. 5:**
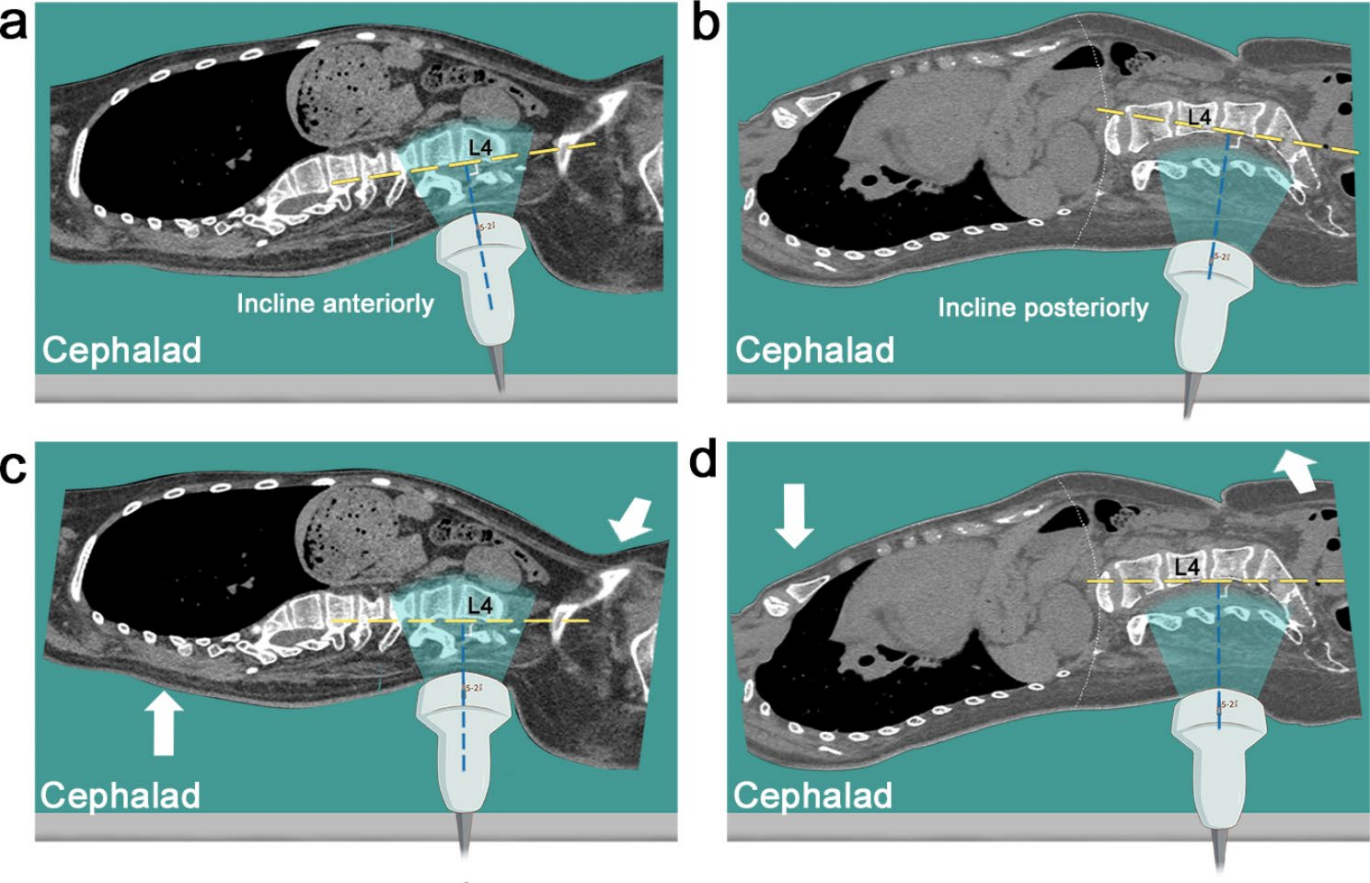
Positioning guided by the orientation of the ultrasound probe. (**a-b**) Sagittal CT image showing the posterior aspect of the scanned vertebra is not parallel to the edge of the bed under the standard lateral decubitus position. Note that a cephalad (**a**) or caudad (**b**) angulation of the probe is commonly required to obtain the optimal visualization of the anterior complex in patients with scoliosis and hyperlordosis. (**c-d**) Sagittal CT image demonstrating modified position with the posterior aspect of the scanned vertebral body adjusted to align with the edge of the bed, allowing for a perpendicularly (to the edge of the bed) placed probe

Different from the conventional method described in the textbook, the needle is inserted perpendicular to the edge of the bed instead of the patient’s back, as the posterior aspect of the target spine is not always aligned with the edge of bed as indicated by the cephalad or caudad angulation of the probe. To obtain optimal visualization of the AC, the sonographer should attempt to keep the direction of the beam as close to perpendicular to the posterior aspect of the vertebral body as possible. Alterations in the curvature of the lumbar spine such as hyperlordosis can cause changes in the orientation of the vertebral bodies being scanned (Fig. 5a-b). Even with maximum flexion of the hips and knees, it is not sufficient to flatten out the abnormal lumbar lordosis. Consequently, the orientation of the back as a whole does not coincide with the orientation of target vertebra. When cephalad angulation is required to obtain optimal visualization of AC, the orientation of the target vertebra is anteriorly obliquely relative to the edge of the bed. To eliminate the need of cephalad angulation, the cranial portion of the torso (the shoulders) should be moved forward while the caudal portion (the hips) to be moved backward, and vice versa (Fig. 5c-d).

Although previous axial and sagittal reconstruction CT imaging was utilized to explain our strategies of lumbar puncture procedures, it should be noted that CT is usually performed in the supine position, and as such, the images obtained may not be fully representative of the in vivo scenario. Merely using bedside ultrasound in an in vivo setting can assist in positioning and selecting an appropriate site and approach. For lumbar punctures in patients with spinal deformities, a preprocedural CT scan is not always required in routine clinical practice; however, reviewing the available imaging prior to performing the spinal procedures can forewarn of potential difficulties. In our case series, we reported nine successful ultrasound-assisted lumbar punctures in challenging spine anatomy; however, eight of which were performed on the same patient. We are excited about larger case series and prospective studies to define the superiority of this approach relative to alternative approaches to intrathecal access for patients with difficulties.

In conclusion, this ultrasound-assisted paramedian horizontal and perpendicular approach may be a promising technique that can help circumvent challenging anatomy.

## Data Availability

All data related to this case report are contained within the manuscript.

## List of abbreviations

3D: three-dimensional
AC: anterior complex
CT: computed tomography
LP: lumbar puncture
PSO: paramedian sagittal oblique
SMA: spinal muscular atrophy
SP: spinous process
